# Ferulic Acid Induces Autophagy and Apoptosis in Colon Cancer CT26 Cells via the MAPK Pathway

**DOI:** 10.3390/molecules28166014

**Published:** 2023-08-11

**Authors:** Shanbin Chen, Dong Zhao, Chunguang Luan, Jia Zheng, Wei Liu, Zheng Feng, Ruiqi Luo, Xinglin Han, Deliang Wang

**Affiliations:** 1 China National Research Institute of Food and Fermentation Industries, Beijing 100015, China; chenshanbin123@163.com (S.C.);; 2 Yibin Wuliangye Co., Ltd., Yibin 644000, China; 3 International Joint Research Center of Quality and Safety of Alcoholic Beverages, Beijing,100015, China

**Keywords:** ferulic acid, apoptosis, autophagy, MAPK pathway

## Abstract

Ferulic acid (FA) is a bioactive compound found in traditional Chinese herbal medicine; for example, it is present in Xinjiang Ferula, but also in strong-flavor Chinese baijiu. FA has been shown to play a crucial role in treating oxidative stress, skin whitening, and eye diseases. In this study, the potential role of FA as a means of inducing apoptosis and inhibiting colon cancer induced by the transplantation of CT26 cells was investigated. The results show that FA adjuvant treatment caused an upregulation in the expression of genes related to autophagy while simultaneously suppressing the expression of inflammatory response elements and improving the bodyweight, glutamic pyruvic transaminase (ALT), and glutamic oxaloacetic transaminase (AST) in vivo. Furthermore, FA inhibited the proliferation of CT26 cells and induced apoptosis, specifically by activating the phosphorylation of ERK and JNK to enhance the essential proteins BCL-2 and BAX in the apoptosis pathway. These results suggest that FA could be a promising auxiliary therapeutic agent for the treatment of colon cancer. Further research is needed to better understand the mechanisms underlying the beneficial effects of FA and its synergistic effects with other compounds.

## 1. Introduction

Colon cancer (CC) has emerged as a significant global health concern, with alcohol-induced colon cancer posing a greater risk than liver cancer, as reported by the World Health Organization [1,2,3,4,5]. Colon cancer is the prevailing form of cancer found in the gastrointestinal system [6,7]. While surgery is effective for removing malignant tissue in most patients, it is not always sufficient and often has to be combined with radio- and chemotherapy, which have side effects that can limit their effectiveness [8,9,10]. Incorporating suitable compounds in treatment can help alleviate some of the side effects caused by the cancer itself or by radiation therapy used for treatment.

Phytochemicals are being studied for their potential in cancer prevention and treatment due to their anti-cancer effects while being safe. One such chemical of interest is ferulic acid (FA), a natural plant component [11,12]. FA is a natural compound commonly found in traditional Chinese herbs such as Ferula, Ligusticum chuanxiong, and Angelica Sinensis, and is a common additive in baked goods, meat, dairy products, and Chinese baijiu [13,14,15]. While considerable research supports FA treatment of tumors—recent studies have shown FA to be an effective antioxidant with anti-inflammatory and antitumor properties [16,17,18]—little is known about its inhibitory effects on tumors. Additionally, FA has been found to be a prominent component in strong-flavor baijiu, a popular alcoholic beverage [19,20]. It is unclear, however, whether FA has any effect on drug- or alcohol-induced colon cancer. Overall, while a significant amount of research supports the beneficial effects of ferulic acid derivatives, more research is needed to understand the potential benefits of FA in the prevention and treatment of tumors and other related conditions.

Apoptosis, the programmed and systematic death of cells, eliminates unnecessary or diseased cells in a highly regulated manner. It plays a significant role in individual growth and development and the development of related ailments such as cancer, diabetes, and neurodegenerative diseases [21,22,23]. Cell autophagy is also essential to keep cellular homeostasis; it regulates the pathophysiology of tumors and other diseases [24,25]. Apoptosis and autophagy, while different, are interrelated and interdependent [26,27]. Many studies have shown that these two forms of cell death have interacting and limiting effects and functions [28]. The dynamic balance between them is consequential to the physiological activities of cancer cells [29,30]. Excessive autophagy involving intracellular protein and organelle consumption can harm cellular survival, leading to cell death [31]. Therefore, examining the mechanisms of FA on apoptosis and autophagy in detail can help clarify its antitumor attributes and specific mechanisms.

In this study, we investigated the protective effects of FA adjuvant therapy in vitro, via examining the viability of CT26, as well as in vivo, against tumor damage induced by CT26 in Balb/c mice. The results revealed that FA can decrease tumor invasion in the colon by promoting autophagy levels and promoting the expression of essential proteins related to cell apoptosis. Furthermore, FA treatment was found to induce cell apoptosis in CT26 cells and regulate the critical MAPK signaling pathway. This is a significant step towards developing functional foods for colon cancer prevention.

## 2. Results

### 2.1. Effects of FA on the Viability of CT26 Cells

**Figure 1:**
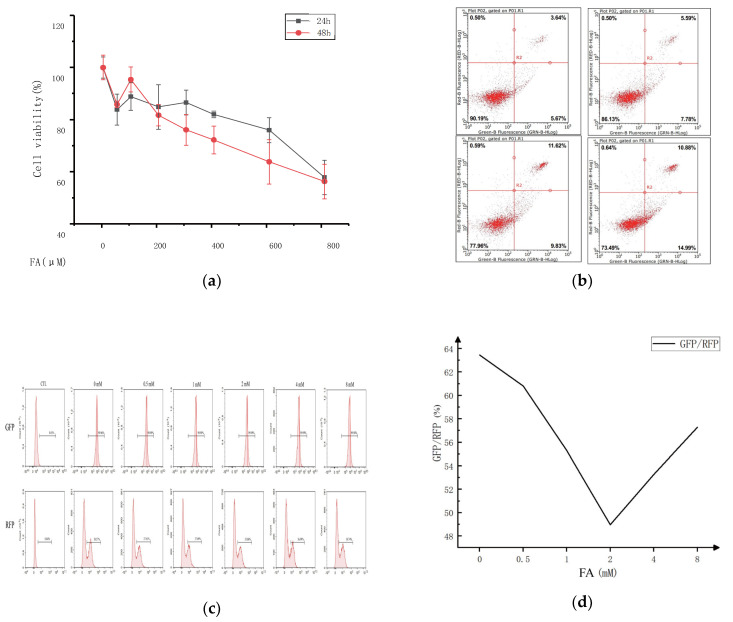
(**a**) CT26 cells were exposed to various concentrations of FA (0–800 μM) for 24 and 48 h, and cell viability was determined using the CCK-8 assay. Data are reported as mean ± S.D. (*n* = 3) for each group. (**b**) The CT26 cells were treated with different concentrations for 24 h and Detection of apoptosis by flow cytometry. (**c**) A lentivirus was used to stably transfer GFP-LC3-RFP-LC3 into cells. The impact of varying concentrations Ferulic acid on cell autophagy was assessed using flow cytometry. (**d**) Quantitative flow cytometry results following the administration of Ferulic acid to cells.

This study examined the impact of varying concentrations of ferulic acid on cell viability to determine the optimal concentration for cell treatment. Figure 1 displays the research findings. Compared to the control group, the activity of the CT-26 cells in each group was significantly suppressed after being pretreated with different ferulic acid concentrations for 24 or 48 h, with the inhibition rate varying depending on the concentration and time. Higher concentrations of ferulic acid resulted in lower cell survival rates, and more extended treatment periods also decreased cell survival. However, treatment with 100 μM of ferulic acid promoted the proliferation of CT-26 cells to some extent, regardless of whether the treatment lasted for 24 or 48 h. Additionally, there was no significant difference in the survival rate of the CT-26 cells for treatments with 800 μM of ferulic acid for either 24 or 48 h. The results show that the IC50 value for ferulic acid with respect to the 24 and 48 h treatments was 800 μM, and concentrations of ferulic acid ranging from 0 to 400 μM had no significant effect on cell proliferation. Therefore, the most effective concentration of ferulic acid for cell treatment is 0–400 μM. But the cell viability decreased with the increase of FA concentration, so the FA could inhibit CT26 cells.

The effect of ferulic acid concentration on the cell viability was investigated using annexin V/propidium iodide (PI) double staining. The fluorescence intensity of annexin V (*X*-axis) and PI (*Y*-axis) was measured, and the resulting scatter plot was divided into four quadrants. The right side (Q2/3) stands for the cells positive for annexin V staining, while the upper part (Q1/2) indicates the cells positive for PI staining. Necrotic cells were found in the (annexinV FITC)-/PI+ area. Late apoptotic cells were found in the upper right quadrant (Q2), characterized by (annexinV FITC)+/PI+ staining. Early apoptotic cells were observed in the lower right quadrant (Q3), with (annexinV FITC)+/PI- staining. Living cells were in the lower left quadrant (Q4), with (annexinV FITC)-/PI- staining. The apoptosis rate, calculated as the sum of the Q2 and Q3 quadrant percentages, increased from 9.31% to 25.87% with ferulic acid treatment. This result suggests that apoptosis of some cells does not necessarily affect the proliferation of other cells in the same culture.

A fluorescent probe, GFP-LC3-RFP-LC3, was constructed to detect the autophagy flux. It spontaneously cleaves into GFP-LC3 (degraded by autophagy) and RFP-LC3, equal moles of ATG4 protease promoted by compounds that serve as an internal control. Autophagy flux can be estimated using the GFP/RFP fluorescence ratio. To examine whether ferulic acid could affect colon cancer by influencing autophagy and the concentration range of the cells treated with ferulic acid, the GFP fluorescence intensity of the ferulic acid treated group was analyzed. It showed a decreasing trend, showing that ferulic acid promoted cell autophagy. However, the effect was not linear, and there was a clear dose-dependence. At lower concentrations, the apoptosis pathway and cell autophagy complemented each other. As the concentration of ferulic acid increased, autophagy played an increasingly essential role in promoting cancer cell death. However, at higher concentrations, the opposite effect appeared.

### 2.2. FA-Inhibited Growth of CT26 Cells in BALB/c Mice without Toxicity

**Figure 2:**
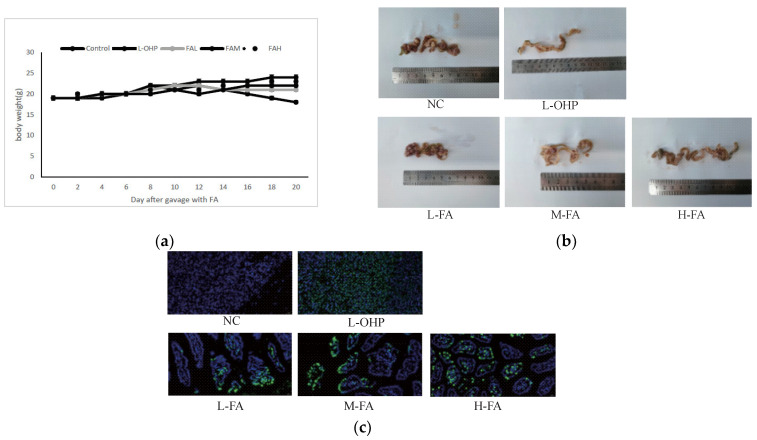
FA inhibited the growth of CT26 cells without displaying observable toxicity in mouse mode. (**a**) When the last day was the 20th day, the mice were treated with FA by oral gavage and were weighed every other day. (**b**) The size of the entire colon tumor was measured on the last day. (**c**) Observe the overall apoptosis of tumor sections by TUNEL at 20 μm. Data are reported as mean ± S.D. (*n* = 3) for each group.

**Figure 3:**
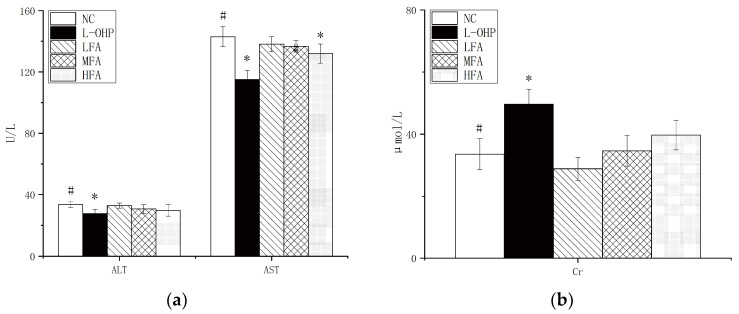

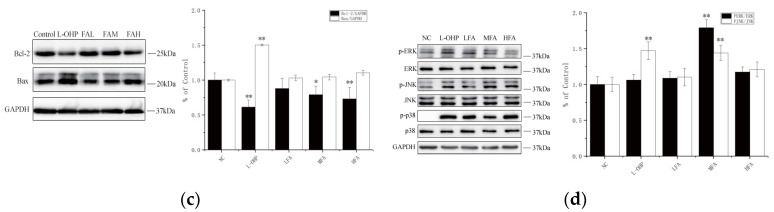
(**a**) Following FA treatment, the mice were sacrificed, blood was collected from the heart, and the levels of alanine aminotransferase (ALT), aspartate aminotransferase (AST), and (**b**) creatinine (Cr) in serum were determined. (**c**) The expression levels of BCL-2 and BAX were detected by Western blotting analysis. Quantification of cleaved BCL-2/GAPDH, BAX/GAPDH. (**d**) The expression levels of p-ERK, ERK, p-JNK, and JNK were detected by Western blotting analysis. Quantification of p-ERK/ERK and p-JNK/JNK. Data are reported as mean ± S.D. (*n* = 3) for each group. * *p* < 0.05, ** *p* < 0.01 vs. normal control group, ^#^ *p* < 0.05 vs.·L-OHP group.

The inhibitory effect of ferulic acid (FA) on the CT26 cells was clear in vitro, prompting an investigation to evaluate its efficacy in vivo. A xenograft model in mice revealed that doses of 40 mg/kg and 80 mg/kg FA significantly reduced the tumor size and weight (Figure 2a). Further evaluation of the drug’s effect on tumor cell apoptosis was conducted using immunohistochemistry. The fluorescence value was used to decide the degree of tumor cell apoptosis, with higher values showing lower tumor cell proliferation. The results showed that 80 mg/kg FA substantially reduced the positive rate of the tumor (Figure 2b,c). To determine whether the FA administration induced toxicity, indicators of liver and kidney function, namely alanine aminotransferase, aspartate aminotransferase, and creatinine, were measured, along with changes in body weight during gavage. FA doses of 20 mg/kg and 40 mg/kg resulted in no significant changes, while 80 mg/kg had a significant effect (Figure 3a,b). In conclusion, FA showed the ability to promote tumor cell apoptosis while being protective to the body.

### 2.3. FA Inhibits Tumor Formation by Promoting the Apoptosis Pathway in BALB/c Mice

To prevent the proliferation and metastasis of cancer cells, the first condition for tumor treatment is to promote apoptosis to inhibit its growth. The apoptosis system is composed of many basic pathways and key proteins, including BCL-2 and BAX. To evaluate the potential effect of FA on apoptosis in CT26 cells, the protein expression levels of BCL-2 and BAX were determined using Western blot. As shown in Figure 3c, after tumor-bearing mice were treated with FA of different concentrations, the level of BAX, a positive correlation protein with apoptosis, was significantly increased, and the Oxaliplatin (L-OHP) was more obvious. At the same time, the level of the protein BCL-2, negatively related to apoptosis, decreased. The semi-quantitative results were consistent with those of the Western blot overall.

In addition, FA processing was used to check the MAPK signal transduction pathway. Abnormalities in this pathway have been proven to be related to many human diseases, including Alzheimer’s disease and cancer. Continuous activation of JNK can mediate neuronal apoptosis; the ERK signaling pathway plays a key role in tumors including cancer cell proliferation, migration, and invasion. Phosphorylation is a requirement for these signaling pathways to exert their specific functions; as shown in Figure 3d, FA treatment gradually increased the activity of JNK and ERK, via increasing their phosphorylation in a dose-dependent manner. These results show that FA can inhibit CT26 colorectal cancer by upregulating the activities of JNK and ERK, thereby promoting apoptosis.

### 2.4. FA Promotes Tumor Autophagy and Inhibits Inflammation

**Figure 4:**
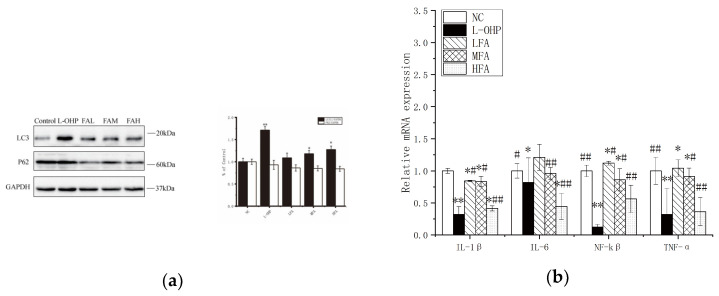
(**a**) The expression levels of LC3 and P62 were detected by Western blotting analysis. Quantification of cleaved LC3/GAPDH and P62/GAPDH. (**b**) The mRNA expression levels of *IL1B*, *IL6*, *NFKB1*, and *TNFA*. Data are reported as mean ± S.D. (*n* = 3) for each group. * *p* < 0.05, ** *p* < 0.01 vs. normal control group, # *p* < 0.05, ## *p* < 0.01 vs.·L-OHP group.

Changes in the autophagy marker LC3 were first detected using Western blotting. The expression of LC3II, which increases when autophagy occurs, was found to have dramatically increased in the L-OHP control after treatment with varying concentrations of FA compared to the normal control group (Figure 4a). The semi-quantitative results were consistent with the immunoblotting results, and the expression of LC3II protein in the high concentration FA treatment group was comparable to that in the L-OHP group, indicating that the effects of FA and L-OHP were comparable. The combination of FA and L-OHP may produce an even greater effect than monotherapy. Previous studies have shown a correlation between autophagy and inflammation, with tumors often partially caused by inflammation. Autophagy has been found to clean up inflammatory responses in most cases. Similarly, in this study, the tumor tissue sections showed an obvious inflammatory reaction (see Figure 4b), and changes in the mRNA expression levels of inflammatory response pathway-related molecules and first responders of inflammation, such as NF-κB, TNF-α, and IL-1β, were detected. The mRNA expression levels of these proteins in the FA group decreased after administration, indicating that the inflammatory effect of cancer was slowed compared to the normal group.

## 3. Discussion

Apoptosis is a form of cell death that is programmed, orderly, and autonomous. Genes control this process, and different cellular stress stimuli, can trigger it. Apoptosis is an important basis for maintaining the stability of organisms. Organisms mainly rely on apoptosis to clear cancer cells. Once the apoptosis is disordered, it will lead to abnormal cell proliferation. The relationship between apoptosis and cancer and the role of apoptosis in cancer treatment has become the focus of antitumor research. In this study, we found that FA pretreatment may reduce the viability of CT26 cells and could promote apoptosis to some extent, as shown via flow cytometry (Figure 1). The incidence rate of colon cancer ranks fourth among the most common malignant tumors in the world, and its mortality is also growing in countries with low incidence rates. In addition to having a direct impact on the body, tumors can also affect appetite to a certain extent, causing weight loss to varying degrees and ultimately leading to continuous deterioration and a decline of the overall health. FA can alleviate the weight loss caused by the tumor and even keep the normal growth of mice to a certain extent (Figure 2). For the tumor itself, FA can directly regulate colon cancer by inhibiting the overall size of the tumor. This regulation is mainly achieved by promoting apoptosis. According to the TUNEL staining of tumor whole sections, FA can effectively promote the overall apoptosis of cancer cells in vivo.

Apoptosis might be accompanied by inflammation and clearance via autophagy. Obviously, with the treatment of FA, the key pathways of apoptosis BAX and BCL-2 are significantly changed, the tumor suppressor-related gene BCL-2 is inhibited, and the apoptosis-promoting gene BAX is enhanced. This change is closely related to the MAPK pathway. JNK and ERK, key proteins of the MAPK pathway, are activated by phosphorylation, and this is even more significantly promoted under the regulation of a medium concentration of FA (Figure 3). The expression of inflammation-related mRNA was significantly changed, which is consistent with the occurrence of tumors, and expression was high in the normal group, while L-OHP and FA could downregulate it to a certain extent. Most importantly, in previous studies, FA promoted autophagy to some extent in vitro. Following intraperitoneal injection of CT26 into mice, the autophagy-related proteins of tumor cells were determined, and the autophagy was observed to be more obvious. However, the influence of autophagy on tumors at various stages was not discussed. It is generally believed that autophagy in the early stage of a tumor provides energy for tumor development but inhibits tumors in the late stage. At present, the specific mechanism of FA on tumor apoptosis and autophagy has not been clarified.

## 4. Materials and Methods

### 4.1. Chemicals and Reagents

CT26WT (ATCC CRL-2638) cells were purchased from Wuhan Punosai Life Technology Co., Ltd. (Wuhan, China). The primary antibodies against cleaved caspase-3, GAPDH, B-cell lymphoma 2 (Bcl-2), Bcl-2-X-associated protein (Bax), microtubule-associated protein 1 light chain 3 beta (LC3), sequestosome-1 (p62), extracellular regulated protein kinases (ERK), phospho-extracellular regulated protein kinases (p-ERK), c-Jun N-terminal kinase (JNK), and phospho- c-Jun N-terminal kinase (p-JNK), as well as horseradish peroxidase (HRP)-labeled secondary antibodies, were purchased from Cell Signaling Technology (Danvers, MA, USA). L-HOP, 99%, and ferulic acid, 99%, were obtained from Aladdin Co., Ltd. (Shanghai, China).

### 4.2. Cell Culture

CT26 cells were cultured in RPMI-1640 medium from Hyclone, Logan, UT, USA, supplemented with 10% fetal bovine serum and antibiotics (100 U/mL penicillin, 100 μg/mL streptomycin), and were incubated in a 5% CO_2_ atmosphere at 37 °C. CT26 cells were exposed to various concentrations of FA, ranging from 0 to 800 µM for 24 or 48 h. The media were replaced every other day.

### 4.3. Cell Viability Assay

CT26 cells were plated 1.2 × 10^4^ cell/well and cultured in 96-well microplates and treated with FA for 24 h and 48 h, as described above. The viability of the treated CT26 cells was determined with the Cell Counting Kit-8 (CCK-8; Dojin Laboratories, Kumamoto, Japan). After the pretreatment, the medium was replaced with 90 µL medium including 10 µL CCK-8 and incubated at 37 °C for 2 h. Following that, the absorbance was measured at a wavelength of 450 nm using a multi-function reader (SpectraMax iD3; Molecular Devices, San Jose, CA, USA).

### 4.4. Methodology for Flow Cytometry

The pMRX-IP-GFP-LC3-RFP-LC3ΔG (RRID: Addgene _84572) plasmid was used as a template to amplify the GFP-LC3-RFP-LC3ΔG fragment, and then the PCR product was digested by NheI/BamHI and connected to the pCDH-RFP-GFP-hLC3B-EF1-Puro (BioVector NTCC Inc) plasmid to obtain the pCDH-GFP-LC3-RFP-LC3ΔG-Puro plasmid. The plasmid was efficiently transferred into cells through lentivirus transduction and showed stable expression. GFP-LC3-RFP-LC3ΔG cells were plated at a density of 1.2 × 10^5^ in each well. After culture for 24 h to allow for cell adhesion, different concentrations of FA were added into each well, and after 24 h of exposure of the cells to the FA, the samples were collected by PBS and analyzed using flow cytometry.

### 4.5. Animal Experimental Design

In this study, male specific-pathogen-free BALB/c mice weighing 18–22 g were obtained from Beijing Vital River Laboratory Animal Technology in China (Beijing). The animals were housed individually in standard cages under regulated temperature (23 ± 2 °C) and humidity (60 ± 5%) with a 12-h light/dark cycle, and they had free access to food and water. The ethical guidelines of the Regulations of Experimental Animal Administration published by the Chinese Science and Technology Committee were followed during the animal studies. The Ethics Committee of Joekai Biotechnology Co. Ltd. in Beijing approved this study (JK (2022)-W-004). After adapting to the environment, the mice were randomly divided into six experimental groups (*n* = 8 per group). These experimental groups were the normal control (NC), L-OHP control (L-OHP, 5 mg/mL·kg^−1^ BW/2 days), the low FA (LFA; L-OHP, 2.5 mg/mL·kg^−1^ L with FA at 20 mg/mL·kg^−1^ BW/day), the medium FA (MFA; L-OHP, 2.5 mg/mL·kg^−1^ L with FA at 40 mg/mL·kg^−1^ BW/day), and the high FA (HFA; L-OHP, 2.5 mg/mL·kg^−1^ L with FA at 80 mg/mL·kg^−1^ BW/day) groups. The NC, L-OHP, and three treatment groups were intraperitoneally injected with 1 × 10^4^ CT26 cells on the first day. In contrast, mice in the NC group were administered physiological saline in the same manner. Furthermore, mice in the three treatment groups were given the respective doses of FA with L-OHP every two days for 20 days. After a 48-h interval following the final gavage, blood samples were obtained from each mouse through ocular collection. Following that, the mice were humanely euthanized using cervical dislocation, and their tumor tissues and sera were collected and carefully stored at −80 °C. Moreover, a part of the tumor tissue was fixed in 10% paraformaldehyde for future histological analysis.

### 4.6. Western Blot Analysis

Cells were washed twice with ice-cold PBS, followed by harvesting and centrifugation at 5000 rpm for 5 min. The cell pellets were then lysed in a lysis buffer (composed of 50 mM Tris–HCl pH 7.5, 150 mM NaCl, 1% Triton X-100, and a complete protease inhibitor cocktail in 2 ug/mL from Suolaibao, Beijing, CHINA) for 10 min. Subsequently, the cell lysates underwent centrifugation at 15,000 rpm for 10 min at 4 °C. The resulting supernatants were collected and combined with sample buffer (from Suolaibao, Beijing, China). Following a 5 min boiling step, the samples were subjected to SDS/polyacrylamide gel electrophoresis (PAGE). The proteins were then transferred onto polyvinylidene fluoride (PVDF) membranes (GE Healthcare, Bucks, UK) using transfer buffer (containing 25 mM Tris base, 190 mM glycine, and 20% methanol). The transferred membranes were blocked at room temperature for 1 h in a mixture of 5% skimmed milk diluted with TBS-T (consisting of 25 mM Tris base, 137 mM NaCl, 2.7 mM KCl, and 0.1% Tween 20, pH adjusted to 7.4). Following blocking, the membranes were incubated overnight at 4 °C with appropriately diluted primary antibodies in final dilution 1000 times in the blocking buffer. Subsequently, the membranes were washed three times with TBS-T and incubated at room temperature for 30 min with a secondary antibody (CST) and were then conjugated with horseradish peroxidase (HRP) using a 1:5000 dilution in blocking buffer. After three more washes, the membranes were visualized using ECL Select Western blotting detection reagent (GE Healthcare) on a ChemiScope 6200 Touch detector (Qinxiang, Shanghai, CHINA).

### 4.7. Quantitative Reverse Transcription-Polymerase Chain Reaction (qRT-PCR) Analysis of the Genes IL1B, IL6, NFKB1, and TNFA

Total RNA was extracted from the tumor using the Eastep^®^ Super Total RNA Extraction Kit, followed by reverse transcription to generate cDNA samples. The cDNA samples were analyzed with an ABI7500 real-time fluorescence quantitative PCR system, using SYBR Green PCR Master Mix as the fluorescent dye. To normalize the results, *GAPDH* was used as an internal reference gene. The relative gene expressions of *IL1B* (encoding *IL-1β*), *IL6*, *NFKB1* (encoding *NF-κB*), and *TNFA* (encoding *TNF-α*) were determined using the 2^−ΔΔCt^ formula. This approach supplies a reliable and accurate method to assess the gene expression levels in mouse tissue.

### 4.8. Statistical Analysis

All data are presented as the mean ± standard deviation of at least three replicates for each prepared sample. Statistical analysis was conducted using SPSS version 23 (IBM, New York, USA). Significance levels were decided using the Bonferroni post-hoc test. A *p*-value lower than 0.05 (*p* < 0.05) was considered statistically significant.

## 5. Conclusions

In summary, the in vivo experiments showed that FA induced autophagy while increasing various stages of apoptosis in CT26 cells. FA also showed the ability to regulate inflammation and implicate inflammation in inducing apoptosis. Furthermore, the in vivo studies proved that FA could inhibit the growth of CT26 cells in mice without any observable toxic effects. Additionally, FA induced the phosphorylation of MAPK pathway-associated proteins such as p-JNK and p-ERK while increasing the expression of Bax. Overall, these findings highlight the potential of FA as a promising phytochemical with anticancer properties that holds great promise as a targeted therapy for colon cancer.

## Data Availability

No new data were created or analyzed in this study. Data sharing is not applicable to this article.
